# Supplementary material for: The genome sequence of the Light Emerald, Campaea margaritaria (Linnaeus, 1761)
Source: Wellcome Open Res. 2023 Oct 18;8:476. [Version 1] doi: 10.12688/wellcomeopenres.20136.1 (PMC10891428; doi:10.12688/wellcomeopenres.20136.1)
Supplement: Peer review history

# Reviewer report: Reviewer response for version 1

**DOI:** 10.21956/wellcomeopenres.22298.r72644

**Published:** 2024-02-16

**Authors:** Jaakko L.O. Pohjoismäki

**Affiliations:** 1 Department of Environmental and Biological Sciences, University of Eastern Finland, Joensuu, Finland

I have no reservations regarding the presented work. The genome assembly and annotation follows the DToL pipeline. The species is a great addition to the growing list of moth genomes. However, it would have been great to have the heterogametic female sex sampled.

Are sufficient details of methods and materials provided to allow replication by others?

Yes

Is the rationale for creating the dataset(s) clearly described?

Yes

Are the datasets clearly presented in a useable and accessible format?

Yes

Are the protocols appropriate and is the work technically sound?

Yes

Reviewer Expertise:

molecular biology, biodiversity genetics, taxonomy, entomology

I confirm that I have read this submission and believe that I have an appropriate level of expertise to confirm that it is of an acceptable scientific standard.

# Reviewer report: Reviewer response for version 1

**DOI:** 10.21956/wellcomeopenres.22298.r72643

**Published:** 2024-02-02

**Authors:** Saskia Wutke

**Affiliations:** 1 Department of Environmental and Biological Sciences, University or Eastern Finland, Joensuu, Finland

This data note reports the genome of the Light Emerald ( *Campaea margaritaria*), a geometrid moth. Genome sequencing, assembly and annotation is based PacBio HiFi, 10X Genomics and Hi-C data. Overall, the dataset is presented clearly and concisely. The methods are state-of-the-art. Yet, reproducibility would benefit from further details concerning the settings of the employed software tools. Moreover, the relevance of this genome report could be explained more in the background section. For example, it is mentioned briefly that the genome can be used for work on invasive agricultural pests but it is not mentioned anywhere if *Campaea margaritaria* falls into this category. Nevertheless, this genome provides a valuable data resource for future research on insect comparative genomics, biodiversity and evolution.

Are sufficient details of methods and materials provided to allow replication by others?

Partly

Is the rationale for creating the dataset(s) clearly described?

Yes

Are the datasets clearly presented in a useable and accessible format?

Yes

Are the protocols appropriate and is the work technically sound?

Yes

Reviewer Expertise:

evolutionary genetics, insect genomics, phylogenomics

I confirm that I have read this submission and believe that I have an appropriate level of expertise to confirm that it is of an acceptable scientific standard.
